# Association between living alone and all-cause mortality of young and middle-aged patients with acute myocardial infarction: analysis of the China Acute Myocardial Infarction (CAMI) registry

**DOI:** 10.1186/s12889-023-17486-7

**Published:** 2024-01-02

**Authors:** Yu Jiang, Jin-Gang Yang, Hai-Yan Qian, Yue-Jin Yang

**Affiliations:** Center for Coronary Heart Disease, Department of Cardiology, National Center for Cardiovascular Diseases of China, State Key Laboratory of Cardiovascular Disease, Fu Wai Hospital, Chinese Academy of Medical Sciences and Peking Union Medical College, 167 Beilishi Rd, Beijing, 100037 China

**Keywords:** Myocardial Infarction, Living alone, Prognosis, All-cause mortality

## Abstract

**Background:**

Lack of social support is a known predictor of the prognosis after acute myocardial infarction (AMI). Although as a common factor associated with social support, there are limited data on long-term prognostic impact of living status in young and middle-aged patients with AMI.

**Methods:**

We analyzed data from the China Acute Myocardial Infarction (CAMI) Registry, consecutive AMI young and middle-aged patients admitted at 108 hospitals in China between January 2013 and September 2014 were included. Eligible patients were assigned to living alone and not living alone groups based on their living status. The primary endpoint was 2-year all-cause mortality. The secondary endpoints included in-hospital mortality and 2-year major adverse cardiac and cerebrovascular events (MACCEs; a composite of all-cause mortality, MI, or stroke). Multilevel logistic and multilevel Cox regression models were used to evaluate the effect of living status on short-term and long-term outcomes.

**Results:**

A total of 8307 consecutive AMI young and middle-aged patients were included, 192 (2.3%) patients were living alone. Of the analyzed patients, living alone was associated with 2-year all-cause mortality and MACCEs among all analyzed patients after multivariate adjustment (adjusted hazard ratio [HR] = 2.171 [1.210–3.895], *P* = 0.009; adjusted HR = 2.169 [1.395–3.370], *P* = 0.001), but not with poorer in-hospital mortality.

**Conclusions:**

The analysis suggested that living alone was associated with both 2-year all-cause mortality and MACCEs in AMI young and middle-aged patients but did not show an extra effect on the in-hospital mortality after covariate adjustment.

**Trial registration:**

Trial registration number: NCT01874691; Registered 31 October 2012.

**Supplementary Information:**

The online version contains supplementary material available at 10.1186/s12889-023-17486-7.

## Background

Existing researches have shown that living alone was related to a lack of social support and increased risk of social isolation [1], which may lead to a range of adverse cardiovascular outcomes [2, 3]. People who lived alone were more likely to increase stress levels, negatively copied with environmental challenges, and changed the pro-inflammatory and hypercoagulable states [4–6]. Acute myocardial infarction (AMI), a serious manifestation of coronary heart disease (CHD), has become a global public health threat and healthcare burden. The high incidence rate and mortality rate caused heavy medical costs and social burdens to the society [7, 8]. Over the past decade, under the influence of economic, cultural, social concepts and other complex factors, the number of individuals living alone is increasing among young and middle-aged people worldwide which is likely to continue [9–11]. A large systematic review and meta-analysis demonstrated that living alone among people under the age of 65 was associated with an increase in mortality [11]. Although previous studies demonstrated a significant association of living alone with mortality after AMI, the included subjects were focused on older people [12–14]. Considering the increasing number of young and middle-aged individuals living alone, more attention should be paid to the impacts of living alone on the short- and long-term outcomes of those patients with AMI.

However, the proportion and age distribution of people living alone in different countries may considerably vary, attributed in part to the diversity of population structure and lifestyle, as well as cultural and economic differences among countries. To date, there was no clear conclusion about the impact of living status on the prognosis of young and middle-aged patients with post-AMI in the eastern Asian population. Based on the China AMI (CAMI) Registry, a prospective, nationwide registry program of acute MI in the real world, the present study aimed to investigate the relationship between residential status and in-hospital mortality and long-term outcomes of young and middle-aged patients hospitalized for AMI across Mainland China.

## Methods

### Study design

The CAMI registry, a prospective, nationwide, multicenter observational study designed to obtain real-world information of patients with AMI (NCT01874691 at https://www.clinicaltrials.gov/) [15], was approved by the institutional review board central committee at Fuwai hospital, national center for cardiovascular diseases of China. All eligible patients provided written informed consent. A total of 108 hospitals covering 31 provinces and municipalities in China participated in the project, and the study included three levels of hospitals (provincial-, prefectural-, and county-level hospitals), assuring a good representation of routine real-world clinical practice of AMI care in China. Staff in each institution was instructed to enroll consecutive patients with AMI within 7 days of acute ischemic symptoms. Final inclusion criteria met the third Universal Definition for Myocardial Infarction (2012). Eligible patients were diagnosed with AMI including ST-segment elevation myocardial infarction (STEMI) and non–ST-elevation myocardial infarction (NSTEMI) within 7 days of acute ischemic symptoms. Final inclusion criteria met third Universal Definition for Myocardial Infarction, including types 1, 2, 3, 4b, and 4c [16]. Type 4a and type 5 AMIs were not eligible for the CAMI registry.

### Study population

Inclusion and exclusion criteria: young and middle-aged patients (18–65 years old) were included who were diagnosed as AMI in the involved hospitals from January 2013 to September 2014. After excluding patients with undefined AMI type, indeterminate living status, and those without any follow-up record, those included patients were classified as either “living alone” or “not living alone” (including living with spouse, living with children, living with parents, and living with others) according to living status.

### Follow-up and outcomes

Clinical follow-up was planned by trained personnel at 30 days, 6, 12, 18, and 24 months. The adverse outcomes and medical records were reviewed and collected by telephone call and electronic medical record reviews. The primary outcome was 2-year all-cause mortality. The secondary outcomes included in-hospital death and major adverse cardiac and cerebrovascular events (MACCEs), a composite of all-cause death, MI, or stroke.

### Study definitions

Onset-to-arrival time is defined as the duration from the onset of AMI symptoms to patient arrival to the hospital [17]. Diabetes can be diagnosed if one of the following conditions are met: (1) In a patient with classic symptoms of hyperglycemia or hyperglycemic crisis, a random plasma glucose ≥ 11.1 mmol/L; (2) Fasting plasma glucose (FPG) ≥ 7.0 mmol/L; (3) 2-h plasma glucose (PG) ≥ 11.1 mmol/L during oral glucose tolerance test (OGTT); (4) A1C ≥ 6.5% (48 mmol/mol) [18]. Hypertension is defined as office systolic blood pressure (SBP) values at least 140mmHg and/or diastolic blood pressure (DBP) values at least 90 mmHg [19]. Hyperlipidemia can be diagnosed if one of the following standards are met: (1) Total cholesterol (TC) ≥ 5.2 mmol/L or triglycerides (TG) ≥ 1.70 mmol/L; (2) Self-reported physician diagnosed hyperlipidemia or taking specific treatment for previously diagnosed hyperlipidemia [20].

### Statistical analysis

Patients’ baseline characteristics and outcomes were compared between living alone and not living alone groups. Continuous variables were expressed as the median and interquartile range and compared using Mann–Whitney U-test. Categorical variables were presented as counts and percentages and compared with the chi-square or Fisher’s exact test. The Kaplan-Meier survival curves were used to graphically present primary and secondary outcomes in patients living alone or living with others, and the differences between groups in cumulative incidence curves were compared using the log-rank test.

Considering that the study included three levels of hospitals (provincial-, prefectural-, and county-level hospitals), multilevel logistic regression model was used to analyze the association between living status and in-hospital mortality, the following variables were initially fitted in the model: demography variables (age, sex, and type of AMI), socioeconomic variables (medical insurance, education level), cardiovascular risk factors (body mass index, smoking, diabetes, hypertension, and prior myocardial infarction). Multilevel Cox proportional hazards regression model was used to calculate the association between living status and long-term outcomes, including all-cause death and MACCEs at 24 months, after adjusting for potential confounders, including demography variables (age, sex, and type of AMI), socioeconomic variables (medical insurance, education level), cardiovascular risk factors (body mass index, smoking, diabetes, hypertension, and prior myocardial infarction), reperfusion therapy (timely reperfusion, untimely reperfusion, and no reperfusion), and medical therapy at discharge (DAPT at discharge, ACEI/ARB at discharge, β-Blockers at discharge, and statins at discharge). The missing quantitative variables were imputed by the mean value, while qualitative variables were imputed by maximum frequency in multilevel logistic regression models and multilevel Cox proportional hazards regression models. All statistical analyses were performed using SAS software (version 9.4) and a 2-sided *P* < 0.05 was considered statistically significant.

## Results

### Baseline characteristics

The present study included 8307 eligible patients with AMI, among whom the mean age was 51.4 (IQR: 46.0-56.6) years, and 87.8% of them were men. Of the 8307 patients, 192 (2.3%) patients were living alone. The comparisons of baseline characteristics between living alone and not living alone group are shown in Table 1. Patients living alone were more likely to be younger compared with those cohabiting (49.5 [IQR: 44.0-55.7] vs. 51.4 [IQR:46.0-56.6], *P* = 0.004). Compared with the not living alone group, living alone patients were more likely paid by themselves other than by rural or urban insurance in medical expenditures created during hospitalization (19.8% vs. 7.9%, *P* < 0.001). Living alone STEMI patients were less likely to receive reperfusion therapy than not living alone ones (73.8% vs. 83.6%, *P* = 0.002). Besides, the rate of cardiac arrest on admission was higher in living alone group than not living alone group (3.1% vs. 1.0%, *P* = 0.013). However, living alone patients had a lower prevalence of diabetes (9.9% vs. 16.6%, *P* < 0.001), hypertension (33.9% vs. 42.9%, *P* = 0.021) and hyperlipidemia (6.3% vs. 8.6%, *P* = 0.002).

**Table 1:** Baseline characteristics of AMI young and middle-aged patients stratified by living status

Variables	Total (*n* = 8307)	Living alone (*n* = 192)	Not living alone (*n* = 8115)	*P* Value
Age, y	51.4 (46.0-56.6)	49.5 (44.0-55.7)	51.4 (46.0-56.6)	0.004
Female (%)	1017 (12.2%)	20 (10.4%)	997 (12.3%)	0.425
Diagnose				0.345
STEMI (%)	6705 (80.7%)	160 (83.3%)	6545 (80.7%)	
NSTEMI (%)	1602 (19.3%)	32 (16.7%)	1570 (19.3%)	
Medical insurance				< 0.001
Urban insurance (%)	4215 (50.9%)	87 (45.3%)	4128 (51.0%)	
Rural insurance (%)	2933 (35.4%)	58 (30.2%)	2875 (35.5%)	
Self-paid (%)	679 (8.2%)	38 (19.8%)	641 (7.9%)	
College education (%)	1033 (12.5%)	25 (13.2%)	1008 (12.5%)	0.707
Body mass index (kg/m^2^)	24.5 (22.9–26.4)	24.5 (22.5–26.6)	24.5 (22.9–26.4)	0.833
Smoking (%)	5203 (62.9%)	127 (66.1%)	5076 (62.8%)	0.339
Diabetes (%)	1361 (16.4%)	19 (9.9%)	1342 (16.6%)	< 0.001
Hypertension (%)	3540 (42.7%)	65 (33.9%)	3475 (42.9%)	0.021
Hyperlipidemia (%)	711 (8.6%)	12 (6.3%)	699 (8.6%)	0.002
Prior myocardial infarction (%)	483 (5.8%)	15 (7.8%)	468 (5.8%)	0.207
Prior heart failure (%)	66 (0.8%)	3 (1.6%)	63 (0.8%)	0.100
Prior PCI (%)	386 (4.7%)	9 (4.7%)	377 (4.7%)	0.956
Prior stroke (%)	444 (5.4%)	7 (3.7%)	437 (5.4%)	0.052
Prior chronic renal insufficiency (%)	57 (0.7%)	3 (1.6%)	54 (0.7%)	0.296
Onset-to-arrival time				0.936
< 3 h (%)	2107 (25.4%)	51 (26.6%)	2056 (25.3%)	
3–6 h (%)	2147 (25.9%)	50 (26.0%)	2097 (25.9%)	
6–12 h (%)	1268 (15.3%)	32 (16.7%)	1236 (15.2%)	
> 12 h (%)	2736 (33.0%)	58 (30.2%)	2678 (33.0%)	
Heart failure on admission (%)	795 (9.6%)	25 (13.0%)	770 (9.5%)	0.238
Cardiogenic shock on admission (%)	216 (2.6%)	6 (3.1%)	210 (2.6%)	0.876
Cardiac arrest on admission (%)	84 (1.0%)	6 (3.1%)	78 (1.0%)	0.013
Killip class III or IV heart failure on admission (%)	387 (4.7%)	13 (6.8%)	374 (4.6%)	0.193
Heart rate on admission (n/min)	76.0 (66.0–87.0)	78.0 (66.0–91.0)	76.0 (66.0–87.0)	0.196
GRACE score on admission				0.110
≤ 108 (%)	2346 (28.2%)	67 (34.9%)	2279 (28.1%)	
109–140 (%)	3996 (48.1%)	81 (42.2%)	3915 (48.2%)	
> 140 (%)	1965 (23.7%)	44 (22.9%)	1921 (23.7%)	
CK-MB (IU/L)	2.3 (1.0-8.9)	1.2 (1.0-29.3)	2.3 (1.0-8.9)	0.715
TnT (ng/mL)	1.1 (0.4–3.6)	3.5 (3.3-4.0)	1.1 (0.4–3.6)	0.499
TnI (ng/mL)	4.3 (0.8–16.1)	4.3 (1.1–10.9)	4.3 (0.8–16.1)	0.281
NT-proBNP (pg/mL)	353.0 (100.0-1046.0)	327.7 (106.5-1334.5)	353.5 (100.0-1041.0)	0.069
First infarct-related artery				0.694
Left main disease (%)	1710 (51.4%)	30 (52.6%)	1680 (51.3%)	
Left circumflex artery (%)	424 (12.7%)	9 (15.8%)	415 (12.7%)	
Right coronary artery (%)	1196 (35.9%)	18 (31.6%)	1178 (36.0%)	
Coronary angiography				0.241
Single-vessel disease (%)	1341 (40.0%)	26 (43.3%)	1315 (39.9%)	
Two-vessel disease (%)	983 (29.3%)	12 (20.0%)	971 (29.5%)	
Three-vessel disease (%)	1032 (30.8%)	22 (36.7%)	1010 (30.6%)	
Reperfusion for STEMI (%)	5561 (83.4%)	118 (73.8%)	5443 (83.6%)	0.002
Reperfusion for NSTEMI (%)	934 (58.7%)	18 (56.3%)	916 (58.8%)	0.773
DAPT at discharge (%)	7697 (95.4%)	181 (96.8%)	7516 (95.3%)	0.319
ACEI/ARB at discharge (%)	4566 (56.6%)	111 (59.4%)	4455 (56.5%)	0.435
β-Blockers at discharge (%)	5733 (71.0%)	121 (64.7%)	5612 (71.2%)	0.059
Statins at discharge (%)	7400 (91.7%)	174 (93.0%)	7226 (91.6%)	0.480

*STEMI *ST-elevation myocardial infarction, *NSTEMI *non-ST-elevation myocardial infarction, *PCI *Percutaneous coronary intervention, *CK-MB *Creatine kinase-MB, *TnT *Troponin T, *TnI *Troponin I, *NT-proBNP *N-terminal pro-B-type natriuretic peptide, *DAPT *Dual antiplatelet therapy, *ACEI/ARB *Angiotensin converting enzyme inhibitor/angiotensin receptor blocker, *GRACE *Global registry of acute coronary events

### In-hospital mortality

There was no difference in the crude rate of in-hospital mortality between living alone patients and not living alone ones (1.0% vs. 1.4%, *P* = 0.674). After being adjusted, the living status-based difference was still not significant (adjusted OR = 0.535 [95% CI: 0.070–4.060], *P* = 0.545) (Table 2).

**Table 2:** Crude and adjusted OR/HR for living status

Treatments	Total (*n* = 8307)	Live alone (*n* = 192)	Not living alone (*n* = 8115)	Unadjusted OR/HR (95% CI)	*P* Value	Adjusted OR/HR (95% CI)^a^	*P* Value
In hospital mortality	116/8307 (1.4%)	2/192 (1.0%)	114/8115 (1.4%)	0.739 (0.181,3.013)	0.674	0.535 (0.070,4.060)	0.545
2-year all-cause mortality	309/8008 (3.9%)	13/181 (7.2%)	296/7827 (3.8%)	1.878 (1.078,3.272)	0.026	2.171 (1.210,3.895)	0.009
MACCEs	559/8306 (6.7%)	23/192 (12.0%)	536/8114 (6.6%)	1.918 (1.251,2.939)	0.003	2.169 (1.395,3.370)	0.001

^a^Multilevel logistic regression model adjusted for demography variables (age, sex, and type of AMI), socioeconomic variables (medical insurance, education level), cardiovascular risk factors (body mass index, smoking, diabetes, hypertension, and prior myocardial infarction). Multilevel Cox regression model adjusted for demography variables (age, sex, and type of AMI), socioeconomic variables (medical insurance, education level), cardiovascular risk factors (body mass index, smoking, diabetes, hypertension, and prior myocardial infarction), reperfusion therapy (timely reperfusion, untimely reperfusion, and no reperfusion), and medical therapy at discharge (DAPT at discharge, ACEI/ARB at discharge, β-Blockers at discharge, and statins at discharge)

Missing qualitative variables were imputed by the highest frequency count, and missing quantitative variables were imputed by the mean value

*CI *Confidential interval, *MACCEs *Major adverse cardiac and cerebrovascular events, *OR *Odd ratio, *HR *Hazard ratio

### Long-term outcome

**Fig. 1:**
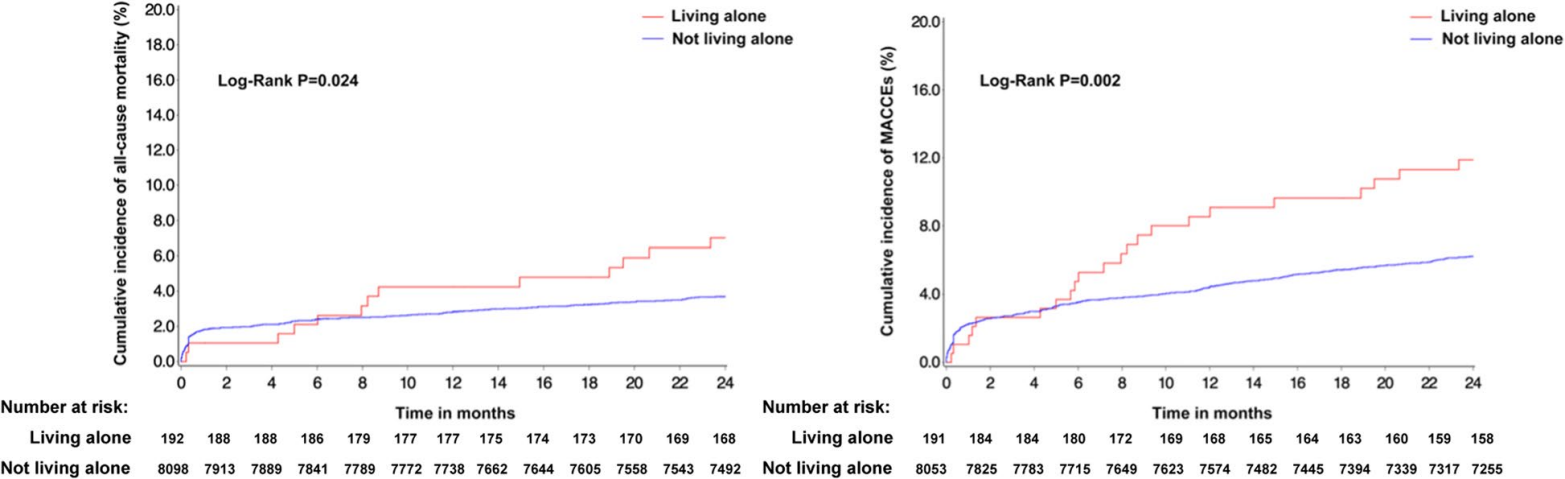
Kaplan-Meier curves for 2-year all-cause mortality and MACCEs

Without adjusting for baseline characteristics, the crude rate of 2-year all-cause mortality and MACCEs was significantly higher in living alone patients than not living alone ones (7.2% vs. 3.8%, *P* = 0.026; 12.0% vs. 6.6%, *P* = 0.003). After covariate adjustment, patients living alone patients had statistically significantly higher rates of both 2-year all-cause mortality (adjusted HR = 2.171 [95% CI: 1.210–3.895], *P* = 0.009) and MACCEs (adjusted HR = 2.169 [95% CI: 1.395–3.370], *P* = 0.001) (Table 2). During a 24-month follow-up, the Kaplan-Meier analysis also indicated that young and middle-aged patients living alone had significantly higher 2-year all-cause mortality and MACCEs risk (Fig. 1).

### Subgroup analyses

**Fig. 2:**
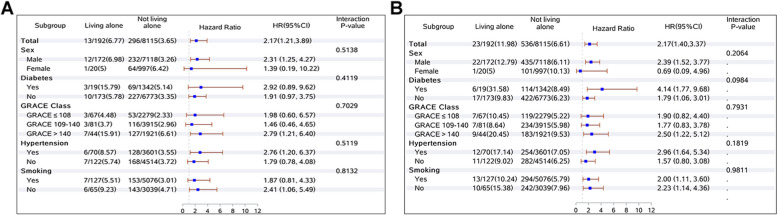
Comparative adjusted hazard ratios of 2-year all-cause mortality (**A**) and MACCEs (**B**) between living alone and not living alone group for each subgroup. HR was adjusted by demography variables (age, sex, and type of AMI), socioeconomic variables (medical insurance, education level), cardiovascular risk factors (body mass index, smoking, diabetes, hypertension, and prior myocardial infarction), reperfusion therapy (timely reperfusion, untimely reperfusion, and no reperfusion), and medical therapy at discharge (DAPT at discharge, ACEI/ARB at discharge, β-Blockers at discharge, and statins at discharge)

With stratification of living status by patient sex, diabetes, GRACE score, hypertension, and smoking, Fig. 2A presented the results from subgroup analyses regarding the association between living alone and 2-year all-cause mortality after adjusting for potential confounding variables. Living alone status significantly increased the risk of 2-year all-cause mortality with respect to man (adjusted HR 2.31, 95% CI 1.25–4.27), GRACE score > 140 (adjusted HR 2.79, 95% CI 1.21–6.40), hypertension (adjusted HR 2.76, 95% CI 1.20–6.37), and no smoking (adjusted HR 2.41, 95% CI 1.06–5.49). However, there was no significant interaction between living status and 2-year all-cause mortality with respect to sex, diabetes, GRACE score, hypertension, and smoking. When performing subgroup analysis for MACCEs, no significant interaction was also observed between living status and MACCEs (Fig. 2B).

## Discussion

In this multicenter, nationwide, prospective Chinese AMI patient registry, the major findings of present analysis are as follows: (1) Compared with not living alone group, young and middle-aged patients with AMI in living alone group had no significant difference in the crude rate of in-hospital mortality. (2) Patients who lived alone were associated with an increased risk of 2-year all-cause mortality and MACCEs compared to those who resided with others. This association remained significant even after adjusting for potential confounding variables. (3) Living alone significantly increased the risk of 2-year all-cause mortality in men, GRACE score > 140, hypertension, and no smoking subgroups.

This is the first study aimed at evaluating the relationship between living status and short-term and long-term outcomes in young and middle-aged patients with AMI. From the baseline characteristics of our cohort, living alone patients were relatively paid by themselves during hospitalization, and higher rate of cardiac arrest on admission than not living group. Given no difference in the onset-to-arrival time between patients living alone and those not living alone, the risk of cardiac arrest upon admission for young and middle-aged patients with AMI who lived alone was increased partly due to the failure to promptly administer cardiopulmonary resuscitation and other emergency medical interventions. In contrast with many stereotypes, before the occurrence of AMI, living alone patients were more likely to have a lower prevalence of cardiovascular comorbidities, such as diabetes, hypertension, and hyperlipidemia. In several previous studies [6, 21, 22], there were also a lower prevalence of diabetes and hyperlipidemia in living alone group, partly due to the small sample size of living alone group, which might influence the objectivity of results. Moreover, in our cohorts, patients who lived with others were generally older. Some studies have indicated that the prevalence of cardiovascular comorbidities in Chinese people increased with age [23–25].

In our present study, there was no remarkable difference in in-hospital mortality rate between patients living alone and those not living alone. Part of the explanation could be ascribed to the fact that young and middle-aged patients with AMI typically did not exhibit obvious comorbidities or significant risk factors for cardiovascular disease. During hospitalization, patients of both living alone and non-living alone received comprehensive and standardized medical monitoring and care. Medical practitioners promptly intervened and managed patients based on their individual conditions, ensuring the provision of appropriate care regardless of their living status. Furthermore, it is worth noting that our study had a limited sample size for in-hospital outcomes, which might underestimate the impact of living status on in-hospital mortality. Although the living status did not reveal a noticeable disparity in in-hospital mortality between living alone and not living alone groups, our present study elucidated a distinct and consistent protective effect of not living alone during long-term follow-up after AMI. Following discharge, patients who did not live alone was associated with a lower 2-year all-cause mortality and MACCEs in comparison to those who lived alone. This association remained significant even after adjustment for potential confounding variables. Several factors have been proposed to explain the association between living alone and adverse long-term outcomes in young and middle-aged patients with AMI. Studies reported associations between living alone and changes in sympathetic activity and increased catecholamines, which activated platelets and macrophages, led to higher levels of interleukin-6 (IL-6), and eventually contributed to development of atherosclerosis and poorer cardiovascular outcomes [26]. After discharge, patients who lived alone were at greater risk of psychological stress and depression, which may aggravate the adverse cardiovascular outcomes caused by these factors via limiting social support and human contact [27]. With the changes in marriage concepts and the high cost of living in modern society, the number of young and middle-aged people living alone has been continuously increasing worldwide. However, due to lacking for care and emotional support of others, people living alone would tend to have less medical supervision and encouragement to maintain a healthy lifestyle, which may be more important in younger patients [28, 29]. Furthermore, younger adults who live alone have greater exposure to unhealthy behaviors, such as smoking, poor diet, and physical inactivity, which might also account for the negative effect [30, 31].

The present results were consistent with several previous studies indicating a significant relationship between living alone and prognostic outcomes after AMI. In the Multicenter Diltiazem Postinfarction Trial, living alone was an independent risk factor for recurrent cardiac events after AMI (HR = 1.54 [95% CI: 1.04–2.29], *P* < 0.03) [13]. Similarly, after adjusting for potential confounding factors, Nielsen et al. [21] found that living alone was an independent predictor of death among employed patients with AMI, with a HR of 2.55 (95% CI: 1.52–4.30). In a study aimed specifically at older women, Norekval et al. [32] demonstrated a higher rate of 10-year mortality after AMI in older women living alone. Furthermore, Schmaltz et al. [6] also found that living alone was independently associated with 3-year mortality with AMI (HR = 1.6 [95% CI: 1.0-2.5]). Compared with woman living alone (HR = 1.2 [95% CI: 0.7–2.2]), man who lived alone had a higher risk of mortality post-discharge (HR = 2.0 [95% CI: 1.1–3.7]).

In our present study, with stratification of living status by patient sex, diabetes, GRACE score stratification, hypertension, and smoking, living alone also significantly increased the risk of 2-year all-cause mortality and MACCEs in men but not in women, indicating that the observed association between living alone and post-AMI prognostic implications also varied by patient sex. Previous study demonstrated that depression could potentially serve as the exclusive mediator linking living alone status to mortality post-AMI. Moreover, there existed a gender disparity between living alone and post-AMI depression. Specifically, men who lived in solitary conditions exhibited a higher propensity for depression [33], which was linked to detrimental cardiovascular outcomes. Besides, living alone might potentially contribute to suboptimal compliance with medication and treatment, as well as inadequate adherence to follow-up recommendations. The impact of this correlation might differ based on the patient’s gender. Unfortunately, our study lacked pertinent data to investigate the potential role of these factor as a confounding or effect-modifying factor. Moreover, considering only 20 female patients in the living alone group, the small sample size may potentially impact the accuracy of the result. Expanding the sample size may obtain more meaningful findings. There was also a significant increase in 2-year all-cause mortality rate among patients living alone with respect to GRACE score > 140 and hypertension. These were likely attributable to the factor that patients living alone generally need self-management of diseases, lack of supervision and reminders from family and friends, and can impede effective control of cardiovascular risk factors such as hypertension and diabetes, which may lead to disease progression and increase the risk of complications.

Although these studies demonstrated the association between living alone and prognosis after AMI [6, 13, 21, 32], several other previous reports did not show independent relationship between living alone and outcomes. For example, date from the Global Use of Strategies to Open Occluded Coronary Arteries (GUSTO) III trial, AMI patients living alone had a higher crude 1-year mortality compared with patients living with others, but there was no significant difference in 1-year mortality rates after adjustment for confounding factors [22]. Emily et al. [27] also found that living alone was not associated with mortality, readmission, or other health status measurements after adjusting for patient and clinical characteristics. Similarly, Berkman et al. [12] found that there was no significant difference in survival in elderly patients living alone versus those living with others after AMI. These different findings may be partially explained by differences in methodology, study sample characteristics, and length of follow-up. For example, the New Haven longitudinal community-based cohort study exclusively enrolled patients with AMI aged 65 years and older, potentially limiting the broader applicability to diverse patients [12]. Additionally, most clinical studies predominantly focused on Western populations [6, 12, 13, 21, 22, 27, 32], inevitably limiting the generalizability of research outcomes to heterogeneous populations.

Currently, studies for AMI mainly focus on the whole or elderly population. With the increased number of young and middle-aged people living alone, great attention should be paid to the impact of the living status on the prognosis with AMI. The present study showed that living with others had a healthy premium on the prognosis of young and middle-aged patients with AMI. Through preventive interventions for young and middle-aged patients living alone, the adverse prognosis gap between living alone and not living alone patients could be reduced.

Nevertheless, there are several limitations in our study. Firstly, the missing or incomplete information and the potential unincluded confounding factors should be considered in the interpretation of results. Secondly, living status is dependent on the description provided by patients or their relatives, which might exist reporting bias. Thirdly, the living alone group in our study is limited in sample size which might influence the validation of our hypothesis. Relevant studies with larger samples are needed in the future.

## Conclusions

The results from our study supported that living alone was independently associated with a substantially increased risk of adverse events during the first 24 months after AMI in young and middle-aged Chinese individuals but did not show an extra in-hospital mortality rate after covariate adjustment. Consideration of living arrangements and household support for living alone young and middle-aged patients with AMI may prevent the poor outcomes.

### Supplementary Information

**Additional file 1: Supplementary Table 1.** Investigators in the CAMI registry.

## Data Availability

The data supporting our findings are available at http://www.CAMIRegistry.org. However, the data is currently not publicly available. Data are available from the corresponding author Yuejin Yang (yangyj_fw@126.com) on reasonable request.

## Abbreviations

CAMI: China acute myocardial infarction
AMI: Acute myocardial infarction
MACCEs: Major adverse cardiac and cerebrovascular events
CHD: Coronary heart disease
STEMI: ST-segment elevation myocardial infarction
NSTEMI: Non–ST-elevation myocardial infarction
FPG: Fasting plasma glucose
PG: Plasma glucose
SBP: Systolic blood pressure
DBP: Diastolic blood pressure
TC: Total cholesterol
TG: Triglycerides
IL-6: Interleukin-6
CK-MB: Creatine kinase-MB
TnT: Troponin T
TnI: Troponin I
NT-proBNP: N-terminal pro-B-type natriuretic peptide
DAPT: Dual antiplatelet therapy
ACEI/ARB: Angiotensin converting enzyme inhibitor/angiotensin receptor blocker
GRACE: Global registry of acute coronary events
