# Supplementary material for: Association between living alone and all-cause mortality of young and middle-aged patients with acute myocardial infarction: analysis of the China Acute Myocardial Infarction (CAMI) registry
Source: BMC Public Health. 2024 Jan 2;24:14. doi: 10.1186/s12889-023-17486-7 (PMC10759749; doi:10.1186/s12889-023-17486-7)
Supplement: Supplementary file 1 — Additional file 1: Supplementary Table 1. Investigators in the CAMI registry. [file 12889_2023_17486_MOESM1_ESM.docx]

**Supplementary materials**

**Association between living alone and all-cause mortality of young and middle-aged patients with acute myocardial infarction: analysis of the China Acute Myocardial Infarction (CAMI) registry**

**Supplementary Table 1 Investigators in the CAMI registry.**

| ID | Hospital | Province/  Municipality | City | Investigator |
| --- | --- | --- | --- | --- |
| 1 | Fuwai Hospital | Beijing | Beijing | Yuan Wu |
| 2 | Beijing Friendship Hospital | Beijing | Beijing | Hongwei Li |
| 3 | Beijing Tongren Hospital | Beijing | Beijing | Changlin Lu |
| 4 | Beijing Daxing Hospital | Beijing | Daxing | Shujun Cao |
| 5 | Beijing Mentougou Hospital | Beijing | Mentougou | Dezhao Wang |
| 6 | Beijing Pinggu Hospital | Beijing | Pinggu | Guanglin Wei |
| 7 | Beijing Yanqing Hospital | Beijing | Yanqing | Jianbing Wang |
| 8 | Shanghai Jiaotong University Ruijin Hospital | Shanghai | Shanghai | Ruiyan Zhang |
| 9 | Shanghai 10th Hospital | Shanghai | Shanghai | Yawei Xu |
| 10 | Shanghai Fengxian Hospital | Shanghai | Fengxian | Zengyong Qiao |
| 11 | Tianjin Medical School General Hospital | Tianjin | Tianjin | Zheng Wan |
| 12 | Tianjin Baodi Hospital | Tianjin | Baodi | Yanjun Cao |
| 13 | Chongqing Medical School 2nd Hospital | Chongqing | Chongqing | Yaohui Yin |
| 14 | Harbin Medical School 1st Affiliated Hospital | Heilongjiang | Harbin | Weimin Li |
| 15 | Qiqihar 1st Hospital | Heilongjiang | Qiqihar | Shuqing Wang |
| 16 | Tailai Hospital | Heilongjiang | Tailai | Gang Ma |
| 17 | Suihua 1st Hospital | Heilongjiang | Suihua | Yongchen Cai |
| 18 | Jilin University 1st Hospital | Jilin | Changchun | Yang Zheng |
| 19 | Tonghua Central Hospital | Jilin | Tonghua | Xuxia Zhang |
| 20 | Huinan County Hospital | Jilin | Huinan | Hongyan Guo |
| 21 | Shenyang Northern Hospital | Liaoning | Shenyang | Xiaozeng Wang |
| 22 | Fushun Central Hospital | Liaoning | Fushun | Ling Sun |
| 23 | Xiuyan County Hospital | Liaoning | Xiuyan | Jianhua Wu |
| 24 | Neimonggu Medical College 1st Affiliated Hospital | Inner Mongolia | Hohhot | Fengying Chen |
| 25 | Chifeng Hospital | Inner Mongolia | Chifeng | Ronghai Man |
| 26 | Aohan Hospital | Inner Mongolia | Aohan | Yanjie Li |
| 27 | Hebei Medcial School 2nd Affiliated Hospital | Hebei | Shijiazhuang | Xianghua Fu |
| 28 | Qinhuangdao 1st Hospital | Hebei | Qinhuangdao | Qingshen Wang |
| 29 | Qinhuangdao 2nd Hospital | Hebei | Changli | Liying Zhang |
| 30 | North-China Oil-administration General Hospital | Hebei | Renqiu | Xiaoli Gao |
| 31 | Changzhou Hospital | Hebei | Changzhou | Yali Hu |
| 32 | Hengshui Hardison Hospital | Hebei | Hengshui | Qun Zheng |
| 33 | Shanxi Cardiovascular Hospital | Shanxi | Taiyuan | Bao Li |
| 34 | Changzhi Hospital | Shanxi | Changzhi | Yuping zhang |
| 35 | Tunliu Hospital | Shanxi | Tunliu | Yaohong Dong |
| 36 | Henan Provincial Hospital | Henan | Zhengzhou | Chuanyu Gao |
| 37 | Linzhou Hospital | Henan | Linzhou | Zhoushun Qin |
| 38 | Changyuan Hospital | Henan | Changyuan | Guorui Hou |
| 39 | Xinxiang Central Hospital | Henan | Xinxiang | Lingling Liu |
| 40 | Yanjin Hospital | Henan | Yanjin | Shifeng Ren |
| 41 | Ye County hospital | Henan | Ye County | Dezhou wang |
| 42 | Pindingshan 2nd Hospital | Henan | Pindingshan | Xianting Luan |
| 43 | Anyang Prefecture Hospital | Henan | Anyang | Hui Liu |
| 44 | Puyang People’s Hospital | Henan | Puyang | Liping Ma |
| 45 | Xi’an Jiaotong University 1st Hospital | Shan’xi | Xi’an | Zuyi Yuan |
| 46 | Weinan Central Hospital | Shan’xi | Weinan | Junnong Li |
| 47 | Weinan Central Hospital | Shan’xi | Weinan | Junnong Li |
| 48 | Jiuquan Hospital | Gansu | Jiuquan | Yaofeng Yuan |
| 49 | Jinta Hospital | Gansu | Jinta | Huide Liu |
| 50 | Ningxia Medical College General Hospital | Ningxia | Yinchuan | Shaobin jia |
| 51 | Wuzhong Hospital | Ningxia | Wuzhong | Xianghong Luo |
| 52 | Qinghai University Affiliated Hospital | Qinghai | Xining | Yin Liu |
| 53 | Qinhai Cardiovascular Hospital | Qinhai | Xining | Pinfa Liu |
| 54 | Xining 1st Hospital | Qinghai | Xining | Xianning Zhao |
| 55 | Hainan Prefectural Hospital of Qinghai | Qinghai | Gonghe | Bao Ma |
| 56 | Xinjiang Medical College 1st Affiliated Hospital | Xinjiang | Urumchi | Yitong Ma |
| 57 | Changji Hospital | Xinjiang | Changji | Mao Wang |
| 58 | Fukang Hospital | Xinjiang | Fukang | Shiming Gao |
| 59 | Urumchi Friendship Hospital | Xinjiang | Urumchi | Hang Lu |
| 60 | Shandong Provincial Hospital | Shandong | Jinan | Lianqun Cui |
| 61 | Taian Central Hospital | Shandong | Taian | Huanyi Zhang |
| 62 | Xintai Hospital | Shandong | Xintai | Hongyan Zhang |
| 63 | Nanjing University Gulou Hospital | Jiangsu | Nanjin | Biao Xu |
| 64 | Jiangsu North Hospital | Jiangsu | Yangzhou | Shenghu He |
| 65 | Xuzhou 1st Central Hospital | Jiangsu | Xuzhou | Qiang Fu |
| 66 | Jiangyan Hospital | Jiangsu | Jiangyan | Shihai Shen |
| 67 | Anhui Provincial Hospital | Anhui | Hefei | Likun Ma |
| 68 | Fuyang Hospital | Anhui | Fuyang | Bin Ning |
| 69 | Taihe Hospital | Anhui | Taihe | Jili Fan |
| 70 | Zhejiang University 2nd Affiliated Hospital | Zhejiang | Hangzhou | Yong Sun |
| 71 | Taizhou Enze medical Center | Zhejiang | Taizhou | Lijiang tang |
| 72 | Taizhou Hospital | Zhejiang | Linhai | Danlei Xu |
| 73 | Fujian Medical College Union Hospital | Fujian | Fuzhou | Lianglong Chen |
| 74 | Xiamen Heart Center | Fujian | Xiamen | Yan Wang |
| 75 | Fuqing Hospital | Fujian | Fuqing | Ping chen |
| 76 | Longyan 1st Hospital | Fujian | Longyan | Kaihong Chen |
| 77 | Wuhan Tongji Hospital | Hubei | Wuhan | Daowen wang |
| 78 | Jinzhou 1st Hospital | Hubei | Jinzhou | Shuixian peng |
| 79 | Tianmen 1st Hospital | Hubei | Tianmen | Shuping Wan |
| 80 | Gong’an Hospital | Hubei | Gongan | Laxi Zhang |
| 81 | Central South University Xiangya 2nd Hospital | Hunan | Changsha | Shenhua Zhou |
| 82 | Xiangtan Central Hospital | Hunan | Xiangtan | Jianping Zeng |
| 83 | Xiangxiang Hospital | Hunan | Xiangxiang | Chonglun Zhou |
| 84 | Ya’an Hospital | Sichuan | Ya’an | Haibo zhang |
| 85 | Zigong 1st Hospital | Sichuan | Zigong | Dechao Zhong |
| 86 | Danleng County Hospital | Sichuan | Danleng | Yuquan Xiao |
| 87 | Guangxi Medical College 1st Affiliated Hospital | Guangxi | Nanning | Lang Li |
| 88 | Beihai Hospital | Guangxi | Beihai | Hai Zhu |
| 89 | Hepu Hospital | Guangxi | Hepu | Meisheng Lai |
| 90 | Nanchang University 2nd Affiliated Hospital | Jiangxi | Nanchang | Xiaoshu Cheng |
| 91 | Pingxiang Hospital | Jiangxi | Pingxiang | Junming Ye |
| 92 | Shangli Hospital | Jiangxi | Shangli | Qishou Liu |
| 93 | Guizhou Cardiovascular Hospital | Guizhou | Guiyang | Tianhe Yang |
| 94 | Zunyi 1st Hospital | Guizhou | Zunyi | Zhengqiang Yuan |
| 95 | Honghuagang Hospital | Guizhou | Honghuagang | Chengyuan Zhao |
| 96 | Pan County Hospital | Guizhou | Pan | Xianwen Jiang |
| 97 | Guangdong Provincial Hospital | Guangdong | Guangzhou | Jiyan Chen |
| 98 | Guangzhou Traditional Chinese Medical College 1st Affiliated Hospital | Guangdong | Guangzhou | Wei Wu |
| 99 | Jiangmen Hospital | Guangdong | Jiangmen | Gaoxing Zhang |
| 100 | Heshan Hospital | Guangdong | Heshan | Haiyuan Mai |
| 101 | Kunming Medical College 1st Affiliated Hospital | Yunnan | Kunming | Tao Guo |
| 102 | Yunnan St. John’s Hospital | Yunnan | Kunming | Yi Li |
| 103 | Chuxiong People’s Hosptal | Yunnan | Chuxiong | Xiaoming Liu |
| 104 | Yao’an Hospital | Yunnan | Yao’an | Jinlong Xu |
| 105 | Tibet People’s Hospital | Tibet | Lhasa | Gesang Luobu |
| 106 | Hainan Provincial Hospital | Hainan | Haikou | Bin Li |
| 107 | Sanya Hospital | Hainan | Sanya | Tiansong Wang |
| 108 | Wenchang Hospital | Hainan | Wenchang | Dong Wang |
